# Notes on the Present Status of Nitrous Oxid in Surgery

**Published:** 1910-08-15

**Authors:** Frederic W. Bancroft

**Affiliations:** Denver


### NOTES ON THE PRESENT STATUS OF NITROUS OXID IN SURGERY.

FREDERIC W. BANCROFT, M.D., DENVER.

In response to a request by Dr. Mumford, the Chairman of the Committee on Anesthesia of the American Medical Association, Dr. Charles A. Powers of the committee has asked me to give late data on the use of nitrous oxid in surgery. In doing this it is impossible to omit reference to the excellent papers presented to the Section on Surgery at its session of 1908.

In 1868 Andrews, of Chicago, first used a combination of nitrous oxid and oxygen. It is interesting to note that the French were the first to consider nitrous oxid as an anesthetic for long operations. In 1880 Paul Bert published an article on the use of nitrous oxid in protracted surgical operations. In the same year Boddaert published the report of the commission on a mixture of nitrous oxid and oxygen.[2] In the last ten years it has tended to become more prominent as an anesthetic in long operations. Bevan and Haggard have done much to promote its general use.

Muller,[3] in 1905, investigated nitrous oxid in a very thorough manner. He states that death occurs by apnea in man in from two to four minutes by straight inhalations of nitrous oxid. He asserts that the gas is absorbed through the lungs into the blood, and that it is transported to the central nervous system. The ganglion cells take up some

---

2. Rapport de la Commission chargee de l'examination du travail de M. Lehant conservant l'emploi comme agent anaesthetique d'un melange de protoxide d'azote et d'oxygene sans pression, Bull. de l'Acad. Roy. de med. de Belge, Brussels, 1880.

3. Muller: Die Stickstoffoxydulnarkose. Berl. med. Wchnschr., vi, 178; Charter: Administration of Nitrous Oxid and Oxygen, Brit. Dent. Jour., xxvii, 443.

substances from the blood and there is a physical change in the cholesterin-lecithin mixture, resulting in paralysis of the ganglion and central nerve cells. It affects the colored races more rapidly than the white, and women are more easily affected than men. In the higher latitudes it is less deadly than near the equator.

### PATHOLOGY.

During anesthesia there is a hyperemia of the internal organs and the work of the stomach, intestines and liver is hindered. Muller experimented on animals—dogs and guinea-pigs. He would anesthetize for about thirty miutes and wait twelve to twenty hours and repeat. This might be repeated once or twice more, so that every animal had from one to four narcoses. On examination he found drops of fat in the ganglion cells (this occurs after all anesthetics and soon disappears). Fat collections were found in the cells of the brain capillaries, extending to the larger vessels. These were found in the cells of the intima and media; between the cells masses of fat were found, which thicken the vessel walls. If they undergo involution through degeneration of the cells, however, a scar formation takes place, with rarefaction of the vessel wall, giving rise to aneurisms and brain hemorrhage. The heart was occasionally found dilated after long anesthesia and the heart muscle lost its transverse striation, the fibers were clouded and numerous fat droplets bipolar to the nucleus were found.

*Kidneys.*—The urine was found to contain albumin after narcosis. In the epithelium of the convoluted tubules fat was found in considerable quantities. There was fat metamorphosis also in the tubuli recti, but no fat was found in the glomeruli. There was marked hyperemia of the kidney vessels.

*Liver.*—Fat was observed in large and small droplets, mostly in the periphery of the acini; only slightly in the center; in the periphery there was degeneration and necrosis of the cells. There was marked hyperemia and some hemorrhage of the liver vessels and in the parenchyma.

*Lungs.*—Death occurs by apnea through paralysis of the respiratory center. In early narcosis there is stertorous breathing caused by the larynx. Muller thinks the cyanosis due more to a mechanical hindrance than to a lack of oxygen in the blood and over amount of carbon monoxid. Dilation of the pupils and swelling of the tongue occurred when the anesthesia was too deep. There was a stimulation of mucus and salivary secretions. Fat metamorphosis was observed in the epithelium cells of the bronchial tract, and fat droplets in the alveolar cells.

Holden believes that there is an increased tendency to bleeding in nitrous oxid anesthesia. He saw severe lung hyperemia, and he holds that all patients having any diseases of the lungs, or ill with a hemorrhagic diathesis, are unsuitable for nitrous oxid narcosis.

Hilliard,[4] in 1905, published an article entitled "Meteorological Conditions As Affecting Oxid Anesthesia." He stated:

1. When the barometer is high (30 degrees or over) the induction of gas anesthesia is longer, there is less cyanosis and jactitation, the gas can be pushed to the abolition of the conjunctival reflex without undue cyanosis.

2. When the barometer is low it is useless to try to abolish the conjunctival reflex, because the patient becomes so deeply cyanosed and jactitates so excessively. With an increase in the time of induction anesthesia the duration of available anesthesia also increases, and with a pressure of 30 inches of mercury the anesthesia is quiet and lasts nearly as long again as when it falls below 29 inches. When one gives nitrous oxid during a fall in the barometric pressure cyanosis occurs early and in less than a minute jactitation will become a prominent feature while the pupils will

---

4. Hilliard: Meteorological Conditions as Affecting Nitrous Oxid Anesthesia, Brit. Dent. Jour., xxvi, 598.

only have begun to dilate; this, although the breathing is stertorous and the available anesthesia consequently short.

Bevan[5] has used nitrous oxid rather extensively in major operations. In his paper read at the fifty-eigth session of the American Medical Association in 1907, he discussed the use of this agent and gave a list of the operations in which he has employed it. In a recent communication he states that he is using it constantly in major operations. He says that it is contraindicated in lung conditions.

At the meeting of the surgical section of the American Medical Association last year, Hamburger and Ewing[6] reported their experiments on the effect of nitrous oxid on the blood. They examined the blood for hemoglobin before anesthesia, during anesthesia, immediately after consciousness returned, and twenty-four hours later. They found:

1. An increase of 13 per cent. of hemoglobin during anesthesia.

2. A fall of 6.5 per cent. immediately after the mask was removed.

3. The fall reduced to 1.6 per cent. twenty-four hours later.

They then experimented to find the relation of the erythrocytes to this change in hemoglobin. Their conclusions were the following:

1. There was little change in the relation of the red blood cells to the hemoglobin.

2. The fall in hemoglobin noted immediately after anesthesia was not followed closely by a corresponding fall in the erythrocyte count, but that there was a slight increase of erythrocytes which persisted.

3. There was a slight increase in the hemoglobin contents per cell and no demonstrable change in the number of red blood cells.

---

5. Bevan, Arthur D: Nitrous Oxid as an Anesthetic in Major Surgery The Journal, A. M. A., July 20, 1907, p. 197; Bevan and Favill: Acid Intoxication and Late Poisonous Effects of Anesthetics, The Journal A. M. A., Sept. 2, 1905, p. 691.

6. Hamburger and Ewing: The Blood Changes Incident to Surgical Anesthesia, The Journal A. M. A., Nov. 7, 1907, p. 1586.

They concluded that this showed there was no oligocythemia due to hemolysis and no persisting oligochromia or anemia.

Summarizing the series, Hamburger and Ewing found:

1. The rise and fall of hemoglobin reading observed chemically holds true in experimented animals.

2. The return to normal occurs within two hours after anesthesia.

3. There is no accompanying rise and fall in erythrocytes.

4. This change does not correspond exactly to the hemoglobin change, as shown by the high blood decimal during anesthesia and the low quotient immediately afterward.

5. The percentage volume undergoes little or no change.

6. The specific gravity increases and decreases hand in hand with erythrocyte change.

7. There is a post-narcotic leucocytosis closely following the erythrocyte curve.

William D. Haggard,[7] in his excellent paper of last year, analyzes briefly the signs of nitrous oxid anesthesia as follows:

1. Quick, deep and stertorous respiration, slightly irregular.
2. Increased pulse rate.
3. Dilation of the pupil.
4. Absence of eye reflexes.
5. Fixed eyeballs.

The symptoms due to asphyxia are:

1. Cyanosis in various degrees.
2. Jactitation.
3. Clonic movements of the extremities.

He states that when proportionately too much gas is given respiration is irregular, quick and stertorous; the

7. Haggard, W. D.: Nitrous Oxid Anesthesia, Report of the Anesthetic Committee, The Journal A. M. A., Nov. 7, 1908, p. 1576.

eyeballs are turned upwards with the lids widely open and the pupil dilated. Cyanosis is then extreme, the pulse thready and rapid. Continuation of this, with exclusion of air, causes respiratory failure from muscular spasm, although the heart continues to beat.

Haggard classifies the advantages and disadvantages of nitrous oxid anesthesia as follows:

ADVANTAGES.

1. Quickness of action.
2. Absence of uncomfortable sensations to the patient.
3. Almost immediate recovery.
4. Absence of lung complications.
5. Absence of danger to the kidneys.
6. Absence of post-anesthetic vomiting.
7. Improbability of subsequent degeneration of the kidneys, liver and heart.

DISADVANTAGES.

1. It is not suitable for fat, plethoric and alcoholic patients.
2. Its zone of anesthesia is within narrow limits.
3. Lividity and occasional talking occur.
4. It needs an accomplished anesthetist; the apparatus is not always easy to obtain.
5. The cost is not always inconsiderable.

The contraindications for its use are:

1. It should not be used for patients with distended hearts or for those with extremely bad hearts, whether with valvular or myocardial disease.
2. It is not suitable for young children on account of their fear of the mask.
3. It should not be used in cases of narrow or abnormal air passages, enlarged lymph nodes, goiters, or enlarged tonsils and adenoids.

4. It is not useful in the lithotomy position, especially in cases of hemorrhoids, as the patients tend to straighten out their legs.

Buckler[8] uses the apparatus on the same principles as the Hewitt inhaler, with certain modifications suggested by Dr. Joseph Gwathmey.[9] He thinks that the sole objection to nitrous oxid and oxygen is that patients on regaining consciousness are more sensitive to pain than those who are partly dulled for hours as in ether anesthesia. This can usually be overcome by giving a quarter of a grain of morphin hypodermatically just before the gas is administered.

As a preliminary to ether Buckler believes that nitrous oxid has no equal; it prevents the coughing, holding of the breath, swallowing and later, the stage of preliminary excitement.

The contraindications for nitrous oxid anesthesia, according to Ream,[10] are atheromatous and arteriosclerotic conditions; although he thinks that with double diligence and oxygen these cases may be safely anesthetized. He found that sprouting seeds refused to grow when placed in nitrous oxid, but resumed their growth when pure oxygen was added. He is a firm believer in the use of nitrous oxid in obstetrical practice because one may obtain analgesia and even anesthesia without muscular relaxation or disturbance of the normal course of labor. Ream states that uterine contractions are not in the least interfered with when the agent is used in the following method:

On approach of labor pains the patient is permitted to inhale three or four breaths to place her in a state of analgesia; the inhaler is then removed and the process is re-

---

8. Buckler, H. W.: The use of Nitrous Oxid and Oxygen alone, and in combination with Ether as Anesthetics in General Surgery, Maryland, Med. Jour., April, 1908.

9. Gwathmey: Mixed Narcosis, The Journal A. M. A., March 28, 1908, p. 1022; Nitrous Gas and Oxygen as an Anesthetic Agent, with Notes on the Value of Warming the Vapors of Ether and Chloroform, read before the Academy of Medicine, New York, May 21, 1908.

10. Ream. Nitrous Oxid and Oxygen in General Surgery, The Journal A. M. A., Sept. 12, 1908, p. 907.

peated as often as is necessary. Should it be desired to retard labor a small percentage of chloroform may be added. Owing to the rapidity of the action of the gas and its rapid elimination it stands foremost as an obstetrical adjuvant.

Gwathmey uses the Hewitt inhaler as modified by himself. He gives morphin sulphate before operation and warms the gas. By experiments on animals he has demonstrated that nitrous oxid and oxygen are most valuable for weak, anemic men and the majority of women.—*A. M. A. Journal.*

